# Study of the renal parenchymal volume during the human fetal period

**DOI:** 10.1590/S1677-5538.IBJU.2018.0538

**Published:** 2019

**Authors:** Andre L. Lima Diniz, Nadia C. Pinheiro Rodrigues, Francisco J. B. Sampaio, Luciano A. Favorito

**Affiliations:** 1 Unidade de Pesquisa Urogenital, Universidade do Estado do Rio de Janeiro, UERJ, Rio de Janeiro, RJ, Brasil

**Keywords:** Hydronephrosis, Fetus, Kidney, Parenchymal Tissue

## Abstract

**Objective:**

To evaluate the renal parenchymal area in human fetuses, providing a descriptive analysis on the renal area development by demographic factors during the second gestational trimester.

**Material and Methods:**

We analyzed 84 fetuses (44 males and 40 females), for a total of 168 renal units evaluated in terms of longitudinal length, superior pole width, inferior pole width and thickness. Renal volume was calculated by ellipsoid formula. After renal pelvis dissection, length and width were evaluated; as pelvis is free of urine, we considered thickness as 1mm. Renal pelvis volume was also calculated by ellipsoid formula. Renal parenchymal area was assessed by excluding the volume of the renal pelvis from the total renal volume. We performed the statistical analysis by simple linear regression assessing the association between the variables analyzed with the fetal age.

**Results:**

Gestational age ranged from 12 to 23 weeks post conception. Mean renal parenchymal area of the right kidney was 666.22mm^3^ (45.86 to 2375.35mm^3^) and for the left kidney was 606.76mm3 (68.63 to 2402.57mm^3^). No statistical difference was observed between the sides (p-value = 0.3456) or genders (p-value = 0.07429). Linear regression between renal parenchymal volume and gestational age was positive for right kidney (y = 133.74x-1479.94 / r^2^ = 0.4009) and left kidney (y = 149.53x-1761.59 / r^2^ = 0.4591).

**Conclusions:**

The linear regression analysis indicated that renal parenchymal area correlated significantly and positively with fetal age, weight and crown-rump length with no statistical differences between gender or laterality. These growth curves provide a reference for functional volume of the kidney during fetal period.

## INTRODUCTION

Hydronephrosis is defined as a dilation of the renal pelvis and / or renal calyces and is the most common fetal urinary tract alteration, present in approximately 50% of the reported cases of genitourinary abnormalities (1). Its diagnosis is performed with ultrasound (US) examination, using as a parameter the anteroposterior diameter of the renal pelvis in the transversal plane (APDRP) (2). Antenatal hydronephrosis increases the risk of postnatal pathology (3). Mild to moderate dilatations of the fetal urinary tract are associated with high incidence of postnatal nephropathies with potential clinical effect that need close follow-up and medical care (4).

The Society for Fetal Urology (SFU) defined a subjective graduation system for assessment of hydronephrosis (5). In this system, the status of the calyces is the key while the size of the renal pelvis is less important, and therefore its measurement would not be considered necessary (5). A consensus meeting was convened in 2014 and has attempted to correct that by adding size of renal pelvis into their criteria. The proposed Urinary Tract Dilatation (UTD) Classification System was based on six categories in ultra-sound findings: 1) anterior-posterior renal pelvic diameter (APRPD); 2) calyceal dilation; 3) renal parenchymal thickness; 4) renal parenchymal appearance; 5) bladder abnormalities; and 6) ureteral abnormalities (6).

However, it is known that diagnostic methods with high sensitivity can lead to a large number of false positives (7). Thus, it has been proposed that analyzing the renal parenchymal area could better predict the functional reserve of kidneys (8, 9).

In times of improvement in technology on image diagnosis, radiologists are able to measure one more dimension, making it possible to gauge not only areas, but also volumes; so fetal kidney volumetry can be assessed, and functional reserve of the kidneys could be given in terms of renal parenchymal volume. An accurate assessment of the renal parenchymal volume can be achieved by excluding the volume of the renal pelvis from the total renal volume, so the renal parenchymal volume (RPcV) is the gross volume of the kidney in maximal longitudinal length minus the volume of the collecting system (10). Renal parenchyma volume may be important in terms of prognostication of renal reserve, and renal pelvis diameter may be relevant for early prediction of those who are failing conservative management needing surgical intervention. If the parenchyma is compromised, this is a late sign of kidney obstruction when compensatory mechanisms have failed and so, a low renal parenchymal volume could be associated with increased risk of end-stage renal disease.

The analysis of fetal kidney and pelvis development is well known (11–13). Nevertheless, the parameters of the renal parenchymal volume and its development during the human fetal period have not yet been well defined in the literature. The hypothesis stated in our study is: analysis of the growth curves of the RPcV during the fetal period can provide a reference values for the volume of fetal kidney.

The objective of this paper is to evaluate the renal parenchymal volume in human fetuses, providing a descriptive analysis on the renal parenchymal area development by demographic factors during the second gestational trimester, to provide standard values for prenatal hydronephrosis evaluation during US.

## MATERIAL AND METHODS

This study was carried out in accordance with the ethical standards of the hospital's institutional committee on human experimentation.

We studied 84 fresh human fetuses (44 males and 40 females) ranging in age from 12 to 23 weeks post-conception (WPC), during the period from January 2016 through August 2017, for a total of 168 kidneys. The fetuses were macroscopically well preserved. The fetuses came to our laboratory as a donation of the Obstetric section of our hospital. The fetuses were macroscopically well preserved, showed no signs of malformations and the demise was hypoxia. The gestational age was determined in WPC according to the foot-length criterion. This criterion is currently considered the most acceptable parameter to estimate gestational age (14–16). The fetuses were also evaluated regarding total length (TL), crown-rump length (CRL) and body weight immediately before dissection. The same observer made all the measurements. All the kidneys with anomalies (fusion, rotation, duplication) and renal pelvis dilation were excluded from the study.

After the measurements, the fetuses were carefully dissected with the aid of a stereoscopic lens with 16 / 25X magnification. The kidneys were removed together with the ureters, bladder and genital organs. After kidney dissection, we evaluated the following measurements: renal length, width of the superior pole, width of the inferior pole and renal thickness.

**Figure 1:**
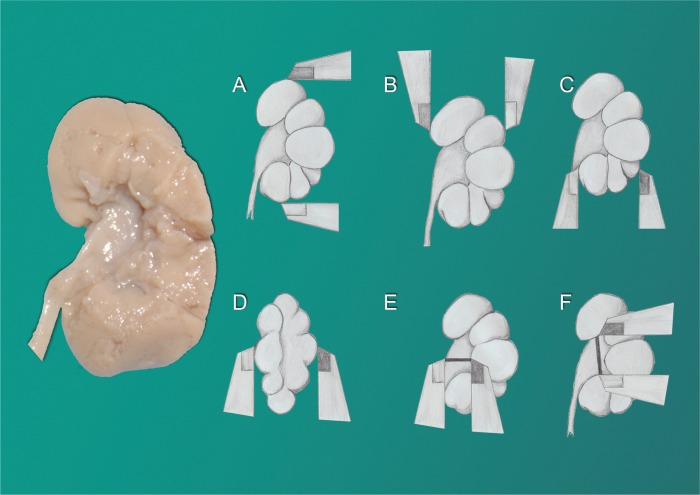
Kidney Measurements. The figure shows a fetal kidney where the renal pelvis and the major calyces were carefully dissected, with removal of the renal parenchyma around the renal pelvis for accurate identification and measurement of these structures; A) Schematic drawing of fetal kidney showing the renal length measurement; B) Schematic drawing of fetal kidney showing the measurement of superior pole width; C) Schematic drawing of fetal kidney showing the measurement of the inferior pole width; D) Schematic drawing of fetal kidney showing the renal thickness measurement; E) Schematic drawing of fetal kidney showing the measurement of the transverse diameter of the renal pelvis (measurement obtained between the distal pelvis extremity and the confluence of the major calyces) and F) Schematic drawing of fetal kidney showing the measurement of the longitudinal diameter of the renal pelvis (distance between the two extremities of the pelvis, i.e., upper-most and lower-most).

After taking the kidney measurements, the renal pelvis and the major calyces were carefully dissected, with removal of the renal parenchyma around the renal pelvis, whenever necessary, for accurate identification and measurement of these structures. The following renal pelvis measurements were taken with the help of a magnifying lens and a digital pachymeter (calibrated in millimeters): transverse diameter of the renal pelvis (measurement obtained between the distal pelvis extremity and the confluence of the major calyces), and the longitudinal diameter of the renal pelvis (distance between the two extremities of the pelvis, i.e., upper-most and lower-most) (Figure-1).

The fetal renal volume was calculated using the ellipsoid formula (10): Renal volume (RV) = renal length x renal thickness x renal width (lower pole + upper pole) / 2 x 0.523.

Since the pelvis was free of urine in the fetuses analyzed, we considered the thickness of the renal pelvis as 1mm, to the fetal renal pelvis volume was calculated using the ellipsoid formula too: fetal renal pelvis volume (RpvV) = renal pelvis length x renal pelvis thickness (= 1mm) x renal pelvis width x 0.523. An accurate assessment of the renal parenchymal volume (RpcV) can be made by subtracting the volume of the renal pelvis from the total renal volume (10, 17).

### Statistical analysis

We performed the statistical analysis using the mean values for each measurement (Weight, TL, CRL and age) by simple linear regression assessing the association between the variables analyzed with the fetal age. In addition, the correlation coefficient (r2) and the p value were obtained for each regression analysis. The correlation coefficient values < 0.4 reflect very weak correlations and the r2 values > 0.7 reflects a strong correlation. p ≤ 0.05 was considered to indicate statistical significance. Software Graphpad Prism 5.0 was used.

## RESULTS

The fetuses presented gestational ages between 12 and 23 weeks post conception (WPC); weighed between 30 and 780g (median = 241.5g; IQR = 135.5) and had crown-rump length between 9.5 and 22.2cm (median = 16cm; IQR = 2.6). The data on fetal anthropometry and kidney measurements are presented in Table-1. The summary of the findings regarding the volume of the kidneys, pelvis and parenchyma for each gender and sides are shown in Table-2.

**Table 1:** The table shows the parameters of renal measurements of the 84 human fetuses analyzed.

	Male fetuses				Female fetuses			
	Right kidney		Left kidney		Right kidney		Left kidney	
	Ẍ	IQR	Ẍ	IQR	Ẍ	IQR	Ẍ	IQR
RV	664.00	546.50	599.50	432.50	745.00	609.74	745.00	607.5
RPvV	8.25	7.45	8.80	7.75	8.15	4.68	8.48	4.11
RPcV	652.01	542.65	592.44	418.87	739.62	607.19	739.36	607.73

**RV** = Renal volume (mm^3^); **RPvV =** Renal pelvis volume(mm^3^); **RPcV =** Renal parenchyma volume(mm^3^); **Ẍ =** Median; **IQR=** interquartile range

**Table 2:** The table shows the parameters of male (M) and female (F) fetuses analyzed.

Age (WPC)	Sex (M/F)	Weight (g)	CRL (cm)	R RV (mm^3^)	L RV (mm^3^)	R RPvV (mm^3^)	L RPvV (mm^3^)	R RPcV (mm^3^)	L RPcV (mm^3^)
12	M	30	9.50	48.50	78.20	2.64	1.87	45.86	76.33
12	M	230	17.00	700.00	754.00	13.28	11.88	686.72	742.12
13	F	60	9.50	90.00	70.00	1.01	1.37	88.99	68.63
13	M	60	10.00	234.00	163.00	3.81	3.52	230.19	159.48
13	M	68	13.00	125.05	184.63	2.48	3.01	122.57	181.62
13	M	76	10.50	195.64	199.81	2.83	4.28	192.81	195.53
13	M	94	13.50	246.07	234.23	4.34	2.74	241.73	231.49
14	M	100	12.40	241.00	275.00	3.13	2.42	237.87	272.58
14	M	104	12.00	327.51	292.11	4.77	7.55	322.74	284.56
14	F	105	12.50	390.00	250.00	4.68	4.10	385.32	245.90
14	F	114	13.00	244.83	259.86	8.28	6.86	236.55	253.00
14	M	134	14.00	266.50	320.20	7.30	7.40	259.20	312.80
15	M	116	14.00	260.56	258.06	7.82	5.07	252.74	252.99
15	M	125	13.30	558.00	479.00	12.20	9.80	545.80	469.20
15	M	165	13.00	450.00	400.00	4.40	5.01	445.60	394.99
15	M	170	13.50	674.00	657.00	8.52	9.40	665.48	647.60
15	F	180	13.50	500.00	500.00	5.47	7.93	494.53	492.07
15	F	180	15.00	440.00	400.00	4.42	6.53	435.58	393.47
15	M	188	16.50	678.20	692.30	22.44	20.73	655.76	671.57
15	M	190	13.00	655.00	591.00	6.74	5.65	648.26	585.35
15	M	206	15.00	808.00	621.50	7.14	17.02	800.86	604.48
16	F	155	14.00	600.00	580.00	14.32	9.70	585.68	570.30
16	M	170	12.50	235.00	334.00	8.20	8.70	226.80	325.30
16	F	170	15.00	360.00	420.00	4.20	9.96	355.80	410.04
16	M	188	15.00	520.40	527.03	8.04	12.01	512.36	515.02
16	F	192	16.00	520.00	380.00	5.95	8.53	514.05	371.47
16	M	195	15.00	786.00	777.00	10.93	7.96	775.07	769.04
16	M	198	17.00	813.00	555.00	12.82	12.75	800.18	542.25
16	F	200	16.00	810.00	830.00	5.86	5.85	804.14	824.15
16	F	220	16.00	1040.00	740.00	8.53	5.78	1031.47	734.22
16	M	230	15.50	686.00	572.00	5.61	6.36	680.39	565.64
16	M	232	15.50	802.00	960.00	12.90	14.76	789.10	945.24
16	M	238	16.00	461.70	698.50	6.51	11.30	455.19	687.20
16	M	245	16.50	585.00	563.00	6.43	7.21	578.57	555.79
16	F	260	15.50	830.00	610.00	9.45	7.83	820.55	602.17
16	F	300	16.20	810.00	750.00	6.94	5.50	803.06	744.50
17	F	112	14.00	270.00	250.00	6.37	6.36	263.63	243.64
17	F	140	14.00	360.00	470.00	8.50	8.43	351.50	461.57
17	F	148	14.50	1180.00	1755.72	3.75	9.81	1176.25	1745.91
17	M	150	14.50	396.00	434.00	2.40	2.97	393.60	431.03
17	F	210	16.00	440.34	482.88	9.27	5.57	431.07	477.31
17	F	225	16.00	670.00	360.00	3.04	5.94	666.96	354.06
17	M	245	15.00	912.00	591.00	3.86	3.30	908.14	587.70
17	M	255	16.00	597.00	608.00	10.80	10.82	586.20	597.18
17	M	260	16.50	582.00	460.00	5.94	6.14	576.06	453.86
17	F	280	16.00	750.00	820.00	7.60	8.53	742.40	811.47
17	M	280	17.00	341.00	544.00	10.21	7.07	330.79	536.93
17	F	290	17.00	990.00	920.00	12.43	11.00	977.57	909.00
17	M	300	17.30	867.00	857.00	9.01	6.65	857.99	850.35
18	F	202	17.50	1150.00	980.00	14.22	20.13	1135.78	959.87
18	F	245	16.00	740.00	940.00	3.15	5.92	736.85	934.08
18	F	280	15.50	940.00	780.00	4.14	5.66	935.86	774.34
18	M	280	16.00	643.00	609.00	11.39	8.90	631.61	600.10
18	M	280	17.50	667.00	523.00	5.34	4.47	661.66	518.53
18	F	285	15.30	1110.00	1040.00	10.11	10.30	1089.89	1029.70
18	M	300	15.00	1089.00	932.00	8.31	7.23	1080.69	924.77
18	F	300	16.50	660.00	620.00	12.55	10.96	647.45	609.04
18	M	335	17.30	979.00	861.00	21.22	23.26	957.78	837.74
18	M	345	18.30	661.00	696.00	13.40	22.75	647.60	673.25
18	M	365	18.50	1175.00	759.00	21.10	12.71	1153.90	746.28
18	M	365	19.00	1485.00	1386.00	23.46	18.60	1461.54	1367.40
18	F	370	17.30	2160.00	1760.00	12.06	6.32	2147.94	1753.68
18	M	370	19.00	1665.00	1466.00	23.95	20.65	1641.05	1445.35
19	F	194	16.50	640.00	680.00	11.13	11.55	628.87	668.45
19	F	250	17.00	700.00	830.00	14.61	24.34	685.39	805.66
19	F	266	17.00	510.00	410.00	8.03	24.76	501.97	385.24
19	F	335	16.50	1080.00	1120.00	7.84	8.80	1072.16	1111.20
19	F	370	17.00	1010.00	1150.00	7.90	9.70	1002.10	1140.30
19	F	380	18.00	1320.00	1270.00	9.70	9.46	1310.30	1260.54
19	F	385	18.50	970.00	1020.00	6.04	8.76	963.96	1011.24
19	F	390	17.00	1840.00	2410.00	6.82	7.43	1833.18	2402.57
20	F	232	16.00	940.00	970.00	7.76	12.41	932.24	957.59
20	F	250	18.00	370.00	410.00	10.44	7.62	359.56	402.38
20	F	304	18.50	390.00	400.00	14.82	17.52	375.18	382.48
20	F	326	17.00	1570.00	1560.00	15.20	12.70	1554.80	1547.30
20	M	390	17.00	1640.00	1698.00	45.07	41.13	1594.93	1656.87
20	M	400	18.50	2094.00	1944.00	12.17	12.73	2081.83	1931.27
20	M	435	19.30	1294.00	1439.00	8.05	12.50	1285.95	1426.50
20	F	455	19.30	1670.00	1670.00	8.64	8.02	1661.36	1661.98
21	F	310	17.00	330.00	460.00	9.33	7.25	320.67	452.75
21	F	314	18.00	1800.00	1860.00	11.80	11.31	1788.20	1848.69
21	M	450	20.00	1493.00	1808.00	40.22	28.21	1452.78	1779.79
22	M	436	19.00	1595.00	1862.00	29.93	36.15	1565.07	1825.85
23	M	780	22.20	2397.00	2416.00	21.65	23.05	2375.35	2392.95

**Wpc =** weeks post conception; **g =** grams; **CRL =** crown rump length; **R RV =** renal volume of right kidney; **L RV =** renal volume of left kidney; **R RPvV =** renal pelvis volume of right kidney; **LRPvV =** renal pelvis volume of left kidney; **R RPcV =** renal parenchymal volume of right kidney; **L RPcV =** renal parenchymal volume of left kidney

The median RV of the right kidney was 676.1mm^3^ (48.5 to 2397mm^3^; IQR = 588.5) and in the left kidney it was 620.8mm^3^ (70 to 2416mm^3^; IQR = 545). No statistical difference was observed among the medians of the samples studies regarding laterality (p = 0.29) and genders (p = 0.34). The median RPcV of the right kidney was 666.22mm^3^ (45.86 to 2375.35mm^3^; IQR = 587.74) and for the left kidney was 606.76mm^3^ (68.63 to 2402.57mm^3^; IQR = 587.74). No statistical difference was observed among the medians of the samples studies regarding laterality (p = 0.4794) and genders (p = 0.329).

**Figure 2:**
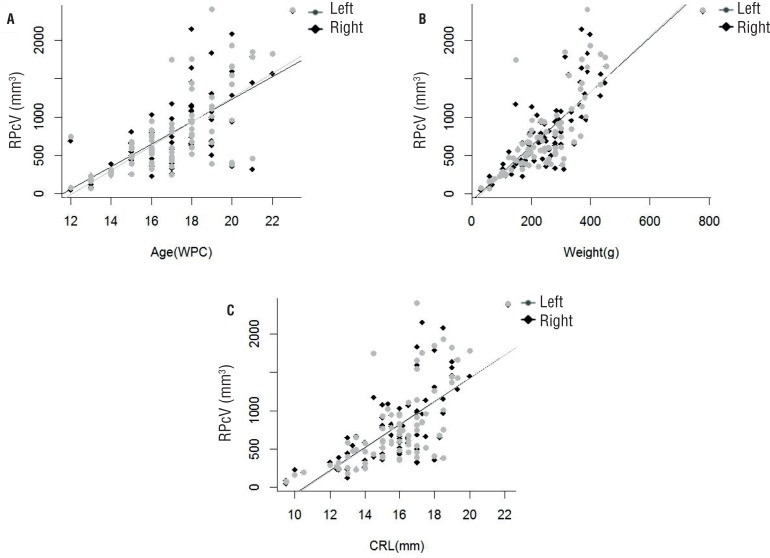
Correlation of the renal parenchymal volume (RPcV) in the left and right side with fetal age, crown-rump length (CRL) and weight during the fetal period studied (13 to 23 weeks post-conception - WPC). The points plotted represent the mean values obtained for each week studied. A) AGE (WPC). Linear regression indicated that RPcV is correlated significantly and positively with fetal age (right side: r^2^ = 4009, p < 0.0001 and left side: r^2^ = 0.4591, p < 0.0001). B) Fetal weight (g). Linear regression indicated that RPcV is correlated significantly and positively with fetal weight (right side: r^2^ = 0.6314, p < 0.0001 and left side: r^2^ = 0.6135, p < 0.0001). C) Crown-rump length (cm). Linear regression indicated that RPcV is correlated significantly and positively with fetal crown-rump length (right side: r2 = 0.4364, p < 0.0001 and left side: r2 = 0.423, p < 0.0001).

We performed simple linear regression analysis between RPcV and RPvV of the right and left kidneys irrespective of gender, correlating them with fetal age, weight and fetal crown-rump length. LC comparing renal parenchymal volume and age, weight and crown-rump length were assessed, so r^2^ values for the correlations of the right kidney parenchymal volumes and age, weight and crown-rump length were 0.46, 0.67 and 0.50 respectively; r^2^ values for the correlations of the left kidney parenchymal volumes and age, weight and crown-rump length were 0.49, 0.64 and 0.46 respectively. LC were also assessed for analysis between renal pelvis volume and age, weight and crown-rump length, so r^2^ values for the correlations of the right kidney pelvis volumes and age, weight and crown-rump length were 0.25, 0.30 and 0.31 respectively; r^2^ values for the correlations of the left kidney pelvis volumes and age, weight and crown-rump length were 0.29, 0.28 and 0.32 respectively (Figure-2).

Although all the correlations were positive, it must be said that r^2^ values less than 0.4 reflect very weak correlations; r^2^ between 0.4 and 0.7 reflects moderate correlations and r^2^ greater than 0.7 reflects strong correlations.

**Figure 3:**
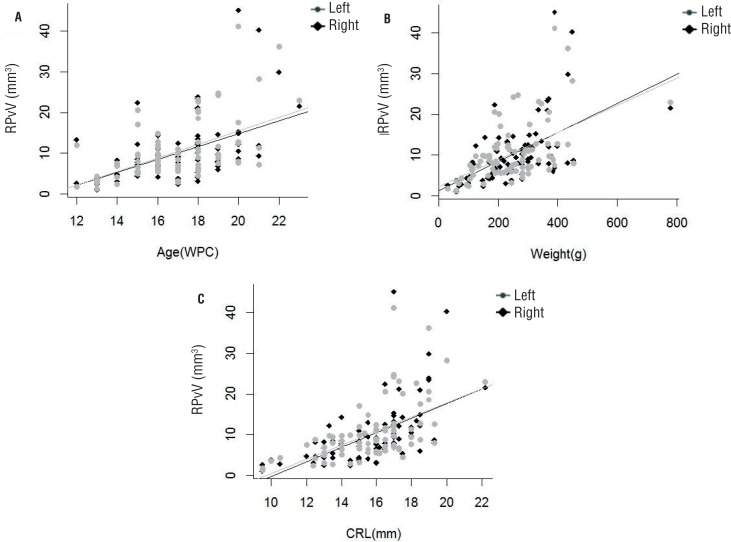
Correlation of the renal pelvis volume (RPvV) in the left and right side with fetal age, crown-rump length (CRL) and weight during the fetal period studied (12 to 23 weeks post-conception - WPC). The points plotted represent the mean values obtained for each week studied. A) AGE (WPC). Linear regression indicated that RPvV is correlated significantly and positively with fetal age (right side: r^2^ = 0.2806, p < 0.0001 and left side: r^2^ = 0.3123, p < 0.0001). B) Fetal weight (g). Linear regression indicated that RPvV is correlated significantly and positively with fetal weight (right side: r^2^ = 0.3054, p < 0.0001 and left side: r^2^ = 0.2833, p < 0.0001). C) Crown-rump length (cm). Linear regression indicated that RPcV is correlated significantly and positively with fetal crown-rump length (right side: r^2^ = 0.3146, p < 0.0001 and left side: r^2^ = 0.3198, p < 0.0001).

Figure-3 shows the correlation graphs and the linear regression of renal pelvis volume related to the fetal parameters studied.

## DISCUSSION

Hydronephrosis with clinical significance has an incidence of 1 / 600, while the prevalence of the hydronephrosis detected during the gestational period is 1 / 50, suggesting that the condition is resolved in many people with development and without the need for treatment (3). Routine prenatal ultrasonography, usually performed at 16 to 20 weeks, has revealed many cases of in utero hydronephrosis (3). According to SFU recommendations, objective parameters have also been used to assess prenatal hydronephrosis (5, 6).

The dilatation of the collecting system has usually been assessed by measuring the APDRP, due to its technical simplicity and reproducibility. However, this measure, which is operator-dependent, may have variations. It has also been observed that in the course of the examination, there may be states of intermittent dilation of the renal pelvis and its collecting system (18). In the second trimester of pregnancy, values greater than 4mm, or in the third trimester values above 7mm, are considered dangerous (5, 18). According to a meta-analysis, the risk of postnatal pathology rises significantly with increasing degree of hydronephrosis (3).

Prediction of future renal deterioration or need for surgical intervention in infants with high-grade hydronephrosis is very important to stratify patients and counsel parents (1, 3, 7). Accordingly with some authors, the precise calculation of renal volume with US using the ellipsoid formula would be inappropriate, since it can underestimate the renal volume, so MRI could be more precise to make these measurements (19, 20); but it has some disadvantages as being more expensive and the need of sedation, so US is still being more usual during the study of prenatal hydronephrosis (3, 5, 6).

A combination of the renal parenchymal volume and APDRP can provide better prediction of postnatal renal function in fetuses with congenital hydronephrosis (21). The new ultrasound techniques, such as three-dimensional (3D) ultrasound using imaging programs, open new perspectives for measuring kidney and renal pelvis volumes. Recent papers show that the RPcV measured by USG is the most promising parameter to evaluate renal disease in fetus with hydronephrosis (22, 23). Kennedy (24) proposed an ultrasound-based fetal renal parenchymal growth curve of normal fetal kidneys from 16 to 38 weeks of gestation. The normal RPcV in children between 0 and 10 months of age was recently demonstrated; the paper of Fischer (9) is very important because the normative measurements of kidney growth assessed could be important to identify those at risk for chronic kidney disease progression.

Some articles have been published about kidney measurements in human fetuses to obtain standard values for obstetrical analysis (11–13). The measurements of renal length and renal pelvis volume can be easily correlated with the fetal parameters during prenatal evaluation (2–4). The renal volume, length and pelvis measurements in human fetuses have been previously studied (11–13), but the RPcV has not been analyzed in human fetuses. This paper presents the first analysis in medical literature of renal parenchymal volume in human fetuses.

In our sample, formed only by fetuses with 23 WPC or less, we did not observe statistically significant differences between any of the renal measurement parameters evaluated between the sexes. We also did not identify statistically significant differences in relation to renal volume parameters when comparing the right and left sides. Various parameters have been proposed to determine the gestational age of human fetuses. Crown-rump length and fetal weight are some of the most important (25, 26). Studies correlating fetal parameters with RPcV during the human fetal period are rare in the literature. This article again is groundbreaking by correlating RPcV and RPvV with fetal weight and CRL. We observed that RPcV and RpvV are also correlated significantly and positively with fetal age, crown-rump length and weight, in the right and left side in both male and female fetuses. These measurements and correlations can be useful for the evaluation of prenatal hydronephrosis as a normative pattern, primarily by US and also by MRI.

We should mention some limitations of this study: 1) small sample size - 168 kidneys are an insufficient number to determine kidney growth with sufficient accuracy. The access to human fetuses is limited, so observations of this sample of 84 human fetuses may be important despite small sample; 2) unequal samples by WPC distribution in the period studied. Nevertheless, the sample distribution during this important period of kidney development was adequate in our opinion; 3) the absence of urine in renal pelvis has led us to assume pelvis thickness as 1mm, we understand that urine volume and pelvic distension / diameter is a dynamic measurement that varies and fluctuates when measured with ultrasound, but as an anatomical study this standardization was the key to solve that limitation. 4) It would be interesting to see if there is a correlation between our post-mortem measurements and parenchymal volume measurements taken by fetal MRI or US (either 2-D or 3-D) with calculations of renal parenchyma area as previously described. This would account for the urine effect in the renal pelvic volume measurement, but due to technical difficulties this evaluation was not possible in our sample.

## CONCLUSIONS

The renal parenchymal volume had a moderate positive correlation with fetal age, crown-rump length and weight in human fetuses in the second gestational trimester. The growth curves of renal parenchymal volume can provide a reference for the functional volume of the kidneys during the fetal period and would give the control to non-invasive image measurements estimating these values.

Studies with the more accurate imaging methods available today could be performed with a larger sample of individuals in an attempt to assess the feasibility of the use of renal parenchymal volume analysis as a method of fetal evaluation in the context of hydronephrosis.

## ABBREVIATIONS

WPC: = Weeks post conception
CRL: = crown rump length
L: = longitudinal length
SW: = superior pole width
IW: = inferior pole width
T: = thickness
RPL: = renal pelvis length
RPW: = renal pelvis width
RV: = renal volume
RPcV: = renal parenchymal volume
RPvV: = renal pelvis volume
APDRP: = antero-posterior diameter of the renal pelvis
SFU: = Society for Fetal Urology
UTD: = Urinary tract dilatation
US: = ultrasound
MRI: = magnetic resonance imaging
Ẍ: = Median
IQR: = interquartile range
LC: = Linear correlation
r^2^: = correlation coefficient
